# Chronic kidney disease in primary care: risk of cardiovascular events, end stage kidney disease and death

**DOI:** 10.1186/s12875-023-02077-7

**Published:** 2023-06-21

**Authors:** Rikke Borg, Margit Kriegbaum, Mia Klinten Grand, Bent Lind, Christen Lykkegaard Andersen, Frederik Persson

**Affiliations:** 1 Department of Medicine, Zealand University Hospital, Roskilde, Denmark; 2 Institute of Clinical Medicine, University of Copenhagen, Copenhagen, Denmark; 3 The Research Unit for General Practice and Section of General Practice, Department of Public Health, University of Copenhagen, Copenhagen, Denmark; 4 Department of Clinical Biochemistry, Copenhagen University Hospital Hvidovre, Hvidovre, Denmark; 5 Department of Hematology, Copenhagen University Hospital, Copenhagen, Denmark; 6 Steno Diabetes Center Copenhagen, Herlev, Denmark

**Keywords:** Chronic kidney disease, Primary care, eGFR, Kidney function, ESKD

## Abstract

**Background:**

The prevalence of chronic kidney disease (CKD) is increasing globally. Early diagnosis in primary care may have a role in ensuring proper intervention. We aimed to determine the prevalence and outcome of CKD in primary care.

**Methods:**

We performed an observational cohort study in primary care in Copenhagen (2001–2015). Outcomes were stroke, myocardial infarction (MI), heart failure (HF), peripheral artery disease (PAD), all-cause- and cardiovascular mortality. We combined individuals with normal kidney function and CKD stage 2 as reference. We conducted cause-specific Cox proportional regressions to calculate the hazard ratios for outcomes according to CKD group. We explored the associations between kidney function and the outcomes examined using eGFR as a continuous variable modelled with penalised splines. All models were adjusted for age, gender, diabetes, hypertension, existing CVD, heart failure, LDL cholesterol and use of antihypertensive treatment.

**Results:**

We included 171,133 individuals with at least two eGFR measurements of which the majority (n = 157,002) had eGFR > 60 ml/min/1.73m^2^ at index date, and 0.05% were in CKD stage 5. Event rates were low in eGFR > 60 ml/min/1.73m^2^ but increased in those with higher stages of CKD. In adjusted analyses we observed an increase in hazard rates for every outcome with every increment in CKD stage. Compared to the reference group, individuals in CKD stage 4 had double the hazard rate of PAD, MI, cardiovascular and all-cause mortality.

**Conclusions:**

Our data from a large primary care cohort demonstrate an early increase in the risk of adverse outcomes already at CKD stage 3. This underlines the importance of studying early intervention in primary care.

**Supplementary Information:**

The online version contains supplementary material available at 10.1186/s12875-023-02077-7.

## Introduction

Chronic kidney disease is defined by an impaired estimated glomerular filtration rate (eGFR) and is silently prevalent in the adult population [1]. The majority of persons with impaired kidney function will never develop the need for dialysis treatment or kidney transplantation, but they are at increased risk of cardiovascular disease (CVD) [2]. Individuals with eGFR < 30 ml/min/1.73m^2^ are often treated in a specialized nephrological setting, whereas the majority of people with eGFR < 60 ml/min/1.73m^2^ (CKD stage 3) are cared for in general practice.

It is projected that CKD globally will become the 5th most prevalent non-communicable disease in 2040, with a serious health care impact in terms of morbidity and mortality [3] as CKD is associated with increased risk of CVD and other complications.

New treatment modalities are becoming available to prevent and delay the progression of chronic kidney disease in addition to standard treatment [4], which mostly include blockers of the renin-angiotensin-aldosterone system. Recent randomized outcome trials in persons with type 2 diabetes and albuminuria have documented the beneficial effect of the non-steroid aldosterone receptor antagonist finerenone, both for kidney-related [5] and for cardiovascular outcome [6]. Furthermore, in patients with CKD, with and without diabetes, the sodium glucose cotransporter 2 (SGLT2) inhibitor dapagliflozin is now approved for the treatment of CKD, following the positive results of the DAPA-CKD trial [7].

As with many chronic conditions, there is great potential for prevention in general practice, but incidence, prevalence and prognosis in the primary care setting are not well known. We therefore aimed to investigate a large primary care population and document the impact of chronic kidney disease.

## Materials and methods

We performed a retrospective observational database linkage cohort study using data at patient level from national health registers in Denmark. All residents are registered with a unique personal identification number (PIN), which enables person-level linkage across registers. The present study linked the included individuals in the Copenhagen Primary Care Laboratory (CopLab) database with the relevant national registers as mentioned below. The study population consisted of persons followed in primary care in the greater Copenhagen area between 2001 and 2016.

### Data sources

The CopLab database contains all test results, 112 million tests from 1.3 million individuals, performed at the Copenhagen General Practitioners’ Laboratory (CGPL). CGPL was the only laboratory that served the general practitioners and other private practicing specialists from 2000 to 2015 located in the greater Copenhagen area in Denmark. The laboratory was accredited for International Organization for Standardization (ISO) standards ISO17025 (until 2011) and ISO15189 (from 2011).

The national registers include information on age, sex, residence, date of death, migration outside of Denmark etc. The Danish National Patient Register contains information on diagnoses from patient hospital contacts since 1977 [8]. The register includes information on the date of admission and type of diagnosis based on the International Classification of Disease (ICD10 codes were used since 1994).

In the present analysis, we included all individuals registered in the CopLab database with at least two measurements of creatinine requested by a general practitioner (GP) between 1st Jan 2001 and 31 Dec 2015, as this is the period with the most consistent data available in the dataset, as well as allowing for time for follow-up. The population was limited to age equal to or above 40 years at baseline.

The date of the second measurement of eGFR was considered the subject’s index date (baseline). This was implemented to ensure a run-in period of 3 to 18 months to ensure stable conditions and rule out acute changes in eGFR at baseline, in accordance with the KDIGO guidelines [9].

Individuals registered in the CopLab database while on chronic dialysis or with a transplanted kidney at the index date were excluded, as were individuals that migrated from Denmark after only one measurement of creatinine.

### Definitions and outcomes

Kidney function (eGFR) was estimated by the CKD-EPI formula based on serum creatinine and expressed in ml/min /1.73m^2^. Conventional CKD groups are used with eGFR > 60 ml/min/1.73m^2^ as reference. CKD 2–5 is defined by eGFR of 89 − 60 (CKD2), 59 − 30 (CKD3), 29 − 15 (CKD4) and < 15 (CKD5) ml/min/1.73m^2^, respectively. Diabetes was defined as the first occurrence of measurement of plasma or serum glucose ≥ 11 mmol/l or HbA_1c_ ≥ 48 mmol/mol in individuals after the age of 30 in the CopLab database. As our data are taken from a laboratory database, we did not have information on the reason for sampling of creatinine, and therefore we combined individuals with normal kidney function and CKD stage 2 and considered them reference for comparison.

Outcomes were based on the Danish death and causes of death registers (all-cause and cardiovascular mortality) or the diagnoses (ICD10) codes in the Danish National Patient Register; myocardial infarction (I21-I22), stroke (I60-I66, G45), heart failure (HF) (I50), peripheral artery disease (PAD) (I70-I79), dialysis (DZ992, DI770+, JAK10, BJFD2, BJFD20, BJFD21, BJFD22, BJFD23, BJFD24, BJFD25, BJFD26) and kidney transplantation (Z940 + KKAS00, KKAS10, KKAS20). Time to overall mortality was defined as time from the index date until death or censoring. The other events were defined as time to the first occurrence of the event of interest or the competing event of death without the event of interest or censoring. Censoring were migration out of Denmark (but not migration from the Copenhagen area, as outcome can be traced in the national registers) or end of the registries (Dec 31st 2016).

### Biochemical assays

Biochemical analyses were performed as previously described: Creatinine, glucose, total and LDL cholesterol [10, 11] and HbA_1c_ [10, 12]. Urine albumin creatinine ratio was measured in spot urine with the commercially available assay Advia 1650/Advia2400 (Bayer, Siemens, Health Diagnostics, Tarrytown, NY, USA) according to the instructions of the manufacturer.

### Statistical analysis

Event rates for outcomes are presented as the number of events per 100 person-years. To investigate the effect of eGFR on the outcomes we estimated the (cause-specific) hazard ratios (HR) using Cox proportional hazards models. The Cox models for overall mortality, end stage kidney disease (ESKD), stroke, MI, PAD, HF and CVD mortality were adjusted for age (spline), sex, antihypertensive drugs, diabetes, previous CVD and LDL cholesterol (log transformed). Stratification on categorical variables was applied when adequate to improve model fit. Since LDL measurements were missing for many individuals, and we assumed that it was due to a missing at random mechanism, we used multiple imputation by substantive model compatible fully conditional specification [13]. For each outcome, we first fitted 56 imputed datasets based on the imputation models. The imputation models were adjusted for eGFR, age, sex, antihypertensive drugs, diabetes (strata), previous CVD, LDL cholesterol level (log transformed), statin use and triglyceride level (log transformed). We did not perform imputations for other clinical markers (i.e. HbA_1c_) as their association to outcome is not as strong as LDL cholesterol. We then fitted the Cox models for the primary outcomes on each imputed data and gathered the estimated HRs using Rubin’s rule. We further explored the continuous associations between kidney function and events using eGFR as a continuous variable with penalised splines with 95% CI, in an analysis adjusted for age, gender, diabetes, existing CVD, heart failure, total- or LDL cholesterol, use of antihypertensive drugs and albuminuria.

## Results

In the CopLab database we identified 4,238,867 measurements of creatinine in 897,864 individuals. After restricting the population according to our inclusion- and exclusion criteria, we defined a study population of 171,133 individuals (Supplemental Fig. 1) with at least two eGFR measurements. Table 1 describes the characteristics of the study population, with individuals in CKD stage 1 and 2 merged, as compared to those in CKD 3, 4 and 5. As expected, the majority of the population (n = 157,002) was in CKD 1 or 2 at index date, and only a small proportion (0.05%) in CKD stage 5. Median age was higher for those with CKD 3–5 as compared to CKD 1-2. Frequent and clinically important comorbidities (cardiovascular disease, heart failure and diabetes) were more prevalent with increasing CKD stage. Of note, of individuals with CKD 3 and 4 approximately 38% were treated with a renin-angiotensin system inhibitor. Across the entire population only between 3 and 7% had albuminuria measured.

**Table 1:** Characteristics at index date

Variable	Reference (eGFR > 60) (n = 157,002)	CKD 3 (n = 13,218)	CKD 4 (n = 823)	CKD 5 (n = 91)
Female gender	86,851 (55.3%)	8455 (64.0%)	496 (60.3%)	59 (64.8%)
Age (years)	61 (51, 71)	81 (74, 86)	83 (76, 88)	83 (76, 89)
CVD	26,945 (17.2%)	5716 (43.2%)	475 (57.7%)	51 (56.0%)
PAD	4581 (2.9%)	1119 (8.5%)	102 (12.4%)	9 (9.9%)
COPD	6287 (4.0%)	1052 (8.0%)	80 (9.7%)	7 (7.7%)
HF	5314 (3.4%)	2287 (17.3%)	257 (31.2%)	23 (25.3%)
Diabetes	11,592 (7.4%)	1158 (8.8%)	66 (8.0%)	9 (9.9%)
Insulin therapy	2039 (1.3%)	508 (3.8%)	53 (6.4%)	9 (9.9%)
Non-insulin diabetes therapy	11,441 (7.3%)	1237 (9.4%)	79 (9.6%)	7 (7.7%)
Aspirin	24,912 (15.9%)	4894 (37.0%)	346 (42.0%)	39 (42.9%)
Clopidogrel	2436 (1.6%)	335 (2.5%)	26 (3.2%)	< 5
Statin	23,718 (15.1%)	2287 (17.3%)	133 (16.2%)	7 (7.7%)
Diuretics	36,877 (23.5%)	7770 (58.8%)	641 (77.9%)	66 (72.5%)
Betablocking agents	19,398 (12.4%)	3231 (24.4%)	226 (27.5%)	27 (29.7%)
Calcium antagonists	20,865 (13.3%)	2955 (22.4%)	240 (29.2%)	28 (30.8%)
Renin angiotensin system blockers	37,174 (23.7%)	4980 (37.7%)	317 (38.5%)	27 (29.7%)
Antihypertensive treatment	84,134 (53.6%)	2767 (20.9%)	102 (12.4%)	11 (12.1%)
Total cholesterol (mmol/l)	5.5 (4.8, 6.2)	5.4 (4.5, 6.2)	5.0 (4.1, 6.0)	5.0 (3.3, 6.3)
*Not measured*	56,462 (36.0%)	7845 (59.4%)	600 (72.9%)	76 (83.5%)
LDL cholesterol (mmol/l)	3.3 (2.6, 3.9)	3.1 (2.4, 3.8)	2.7 (2.2, 3.7)	3.2 (1.6, 3.6)
*Not measured*	85,444 (54.4%)	9933 (75.1%)	696 (84.6%)	82 (90.1%)
Potassium	4.3 (4.1, 4.5)	4.4 (4.1, 4.7)	4.6 (4.3, 5.0)	4.6 (4.3, 5.0)
*Not measured*	35,005 (22.3%)	1687 (12.8%)	76 (9.2%)	9 (9.9%)
HbA_1c_ (mmol/mol)	38.0 (34.0, 45.0)	41.0 (36.0, 50.0)	41.0 (36.0, 47.0)	43.0 (37.0, 50.0)
*Not measured*	110,944 (70.7%)	9851 (74.5%)	622 (75.6%)	62 (68.1%)
Estimated GFR (ml/min/1.73 m^2^)	87.0 (76.0, 98.0)	47.0 (39.5, 53.0)	23.0 (19.0, 26.0)	12.0 (9.0, 13.5)
Urinary albumin creatine ratio (mg/g)	10.0 (10.0, 22.0)	19.0 (10.0, 84.0)	160.0 (32.5, 590.0)	
*Not measured*	145,826 (92.9%)	12,595 (95.3%)	796 (96.7%)	

*All values are median (IQR) or n (%). CKD; chronic kidney disease, CVD; cardiovascular disease, PAD; peripheral arterial disease, COPD; chronic obstructive pulmonary disease, HF; heart failure, LDL; low-density lipoprotein, HbA*_*1c*_; *hemoglobin A1c, GFR; glomerular filtration rate, IQR; interquartile range*

**Fig. 1:**
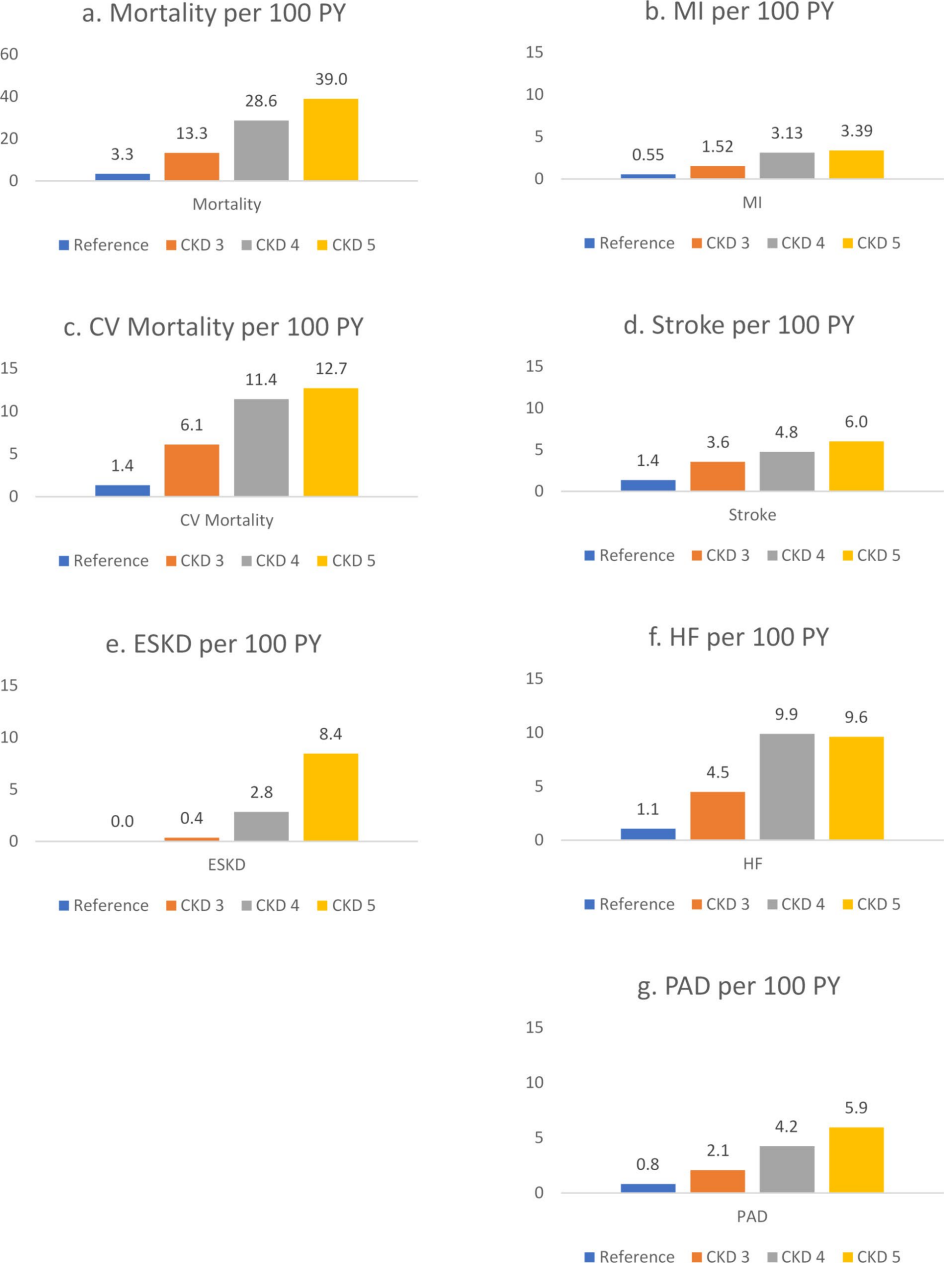
**a-g**. Event rates according to CKD stage per 100 patient-years (PY) CKD; chronic kidney disease, MI; myocardial infarction, CV; cardiovascular, ESKD; end stage kidney disease, HF; heart failure, PAD; peripheral arterial disease.

Figure 1a-g displays the event rates for all outcomes. In general, event rates were low in individuals with CKD stages 1 and 2 and rose in those with higher stages of CKD. Of note, among the cardiovascular outcomes, myocardial infarction had the lowest event rates across all CKD stages as compared with higher rates in PAD, stroke and heart failure, respectively. In those with CKD 3 the heart failure outcome occurred with 4.5 times per 100 person-years (PY) and 9.9 times per 100 PY in CKD4. Mortality rates displayed a marked increase already from CKD 1–2 to stage 3, with rates of 3.3 and 13.3 deaths per 100 person-years of observation, respectively.

**Fig. 2:**
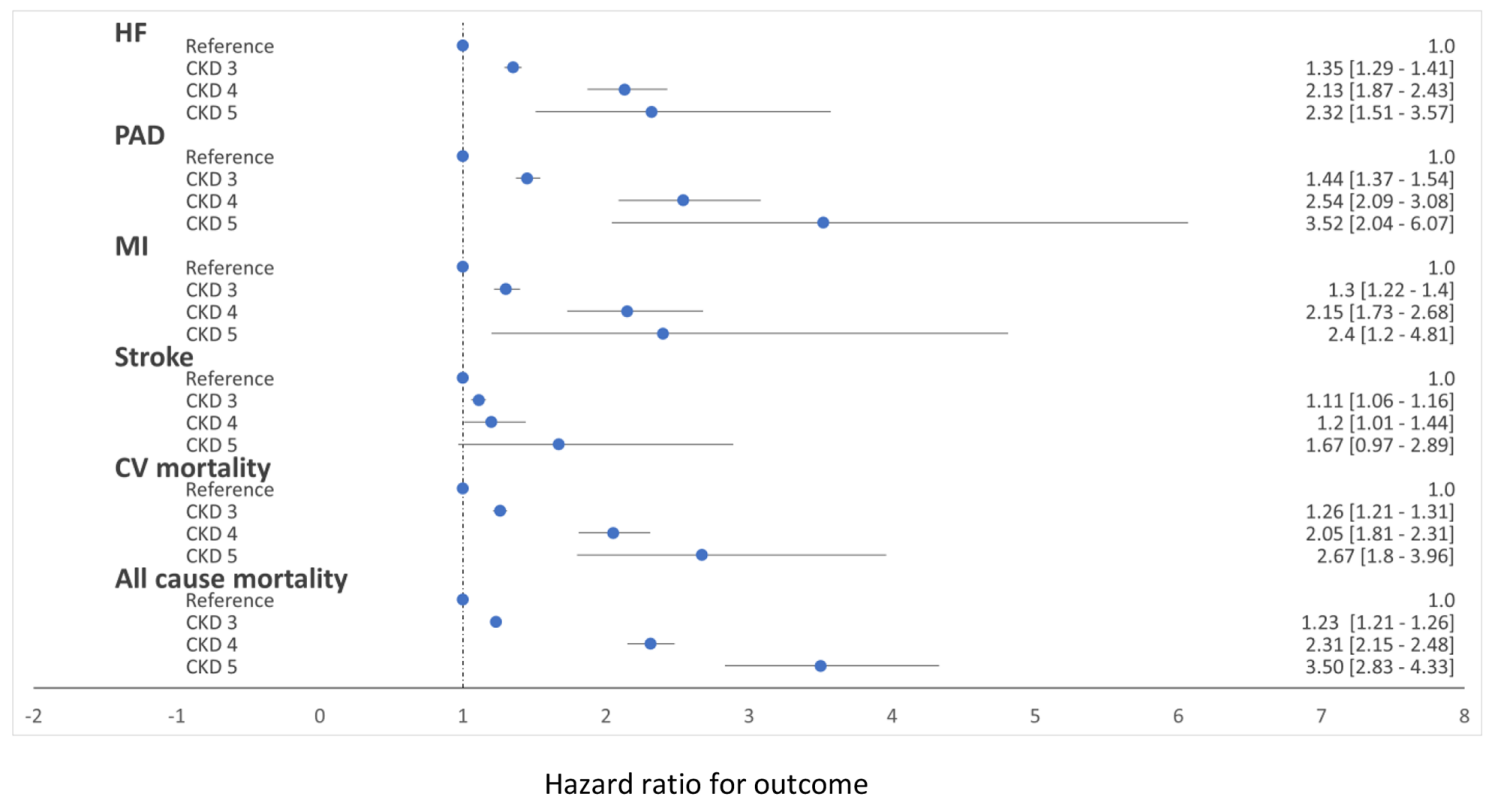
Adjusted hazard ratios according to CKD stage ESKD; end stage kidney disease, CKD; chronic kidney disease, HF; heart failure, PAD; peripheral arterial disease, MI; myocardial infarction, CV; cardiovascular.

In the adjusted analyses we observed an increase in hazard with every increment in CKD stage. Compared to CKD 1 + 2 (eGFR > 60ml/min/1.73 m^2^), individuals in CKD 4 had approximately double the hazard of PAD, MI, CV and all-cause mortality (Fig. 2). As expected, development of ESKD increased dramatically with each CKD stage. Compared to those in CKD 1 + 2, individuals in CKD 3 had a cause-specific hazard ratio (95% confidence interval (CI)) of developing ESKD of 18.3 (15.5–21.7). Likewise, individuals in CKD 4 had a HR of 195 (149–255). The results of the adjusted analyses did not change significantly using the dataset without the imputed LDL-data (data not shown).

The adjusted continuous analyses demonstrate a gradual increase in hazard with declining kidney function for all outcomes. It is particularly evident that the hazard rate of MI, HF, CV death and mortality increases markedly in individuals reaching eGFR of 50 ml/min/1.73 m^2^ and below (Supplementary Fig. 2a-g).

## Discussion

In a large dataset from primary care, we observe an increased hazard of morbidity and mortality in individuals with eGFR < 60 ml/min/1.73m^2^. In addition to the increased risk of kidney failure, the risk of heart failure, peripheral artery disease, myocardial infarction, and cardiovascular death more than doubles when CKD 4 is reached. As a gradual decline in kidney function from CKD 2 to CKD 3 and CKD 4 almost exclusively occurs without symptoms, our findings point to the potential for a wide monitoring of kidney function and earlier cardiovascular risk factor management in a general primary care population. Our large dataset, exclusively from primary care, points to a chronic disease of high prevalence at a time point years before referrals to specialists and as such, adds to previous CKD prevalence studies from diverse populations [14, 15]. Our data can however not support early screening for CKD or early intervention, merely demonstrate that the potential for early detection is there. Randomized trials of screening and early intervention are needed to prove the benefit of such an approach.

Our primary care population comes from a Danish public health care system, which is likely more homogenous than populations in other countries when it comes to socioeconomic status and ethnicity. The adjusted risk of all-cause- and cardiovascular mortality in individuals with CKD 3 was 23% and 26% higher as compared to those with eGFR > 60 ml/min/1.73m^2^. This is similar to findings reported by the CKD Consortium in a large analysis of 30 global populations [16]. The plots of relative hazards for all-cause- and cardiovascular mortality from our study (Supplementary Fig. 2a-b) are similar to the corresponding plots from the CKD consortium, perhaps with a less steep increase in those with CKD 3. Importantly, their analyses included a mix of a general and high-risk population, and also had information on albuminuria levels of many included individuals. Interestingly, the presence of diabetes only had a minor effect on the overall hazard ratios for outcome in the CKD Consortium analysis. Furthermore, Go et al. [2]. found a steep increase in the adjusted hazard ratio for all-cause mortality from 1.8 in individuals with CKD 3 (compared to CKD 1) to 3.2 in those with CKD 4 in their study of > 1.2 million individuals in the Kaiser Permanente Renal Registry. Certainly, there are distinct differences in geography, ethnicity, demography, follow-up, and health care systems between our population and both the abovementioned studies, making a direct comparison difficult. However, as compared to global studies in mixed populations, our findings still point to a potential for prevention and prolonged survival in primary care before the onset of cardiovascular and late-stage kidney disease.

We found higher event rates for HF than for MI in cases with CKD 3-5, however the hazard ratios in the analysis adjusted for comorbidities were similarly elevated in those with CKD 3 compared to eGFR > 60 ml/min/1.73m^2^. This points to the cardiovascular risk in this population, but also demonstrates that heart failure is as important to look for as coronary heart disease. In the Atherosclerosis Risk In Communities (ARIC) study, the authors found that risk of heart failure was doubled in individuals with CKD 3, especially in those with prevalent coronary heart disease [17]. Whether there is a causal link between impaired kidney function and heart failure is much discussed, and may yet be established, but a large part of the risk for heart failure stems from a history with coronary heart disease. Furthermore, both albuminuria and impaired kidney function are known risk markers for outcome in heart failure patients [18, 19]. A general practitioner should therefore acknowledge the extra risk for heart failure when an individual with coronary heart disease presents with CKD 3 or higher. Our eGFR data are collected before the sodium glucose co-transporter 2 (SGLT2) inhibitors were recommended for the treatment of heart failure and for the treatment of chronic kidney disease. Hopefully, the introduction of SGLT2 inhibitors can help lower future risk in individuals with type 2 diabetes, CKD or heart failure or any combination of these conditions.

The reasons for the elevated overall cardiorenal risk in individuals with CKD are many. Traditional risk factors like hypertension, diabetes, smoking and elevated cholesterol levels contribute to both cardiovascular and renal risk, but are all modifiable with well recognized risk reductions. Persistent focus on prevention in primary care should be based on efforts targeting these risk factors. There are, however, indications that the initiation of preventive treatment declines with lower kidney function, as pointed out by Fox et al [20]. They found that patients with CKD admitted with myocardial infarctions had lower use of evidence-based medication at discharge, but also less use of counselling regarding smoking cessation, diet and exercise, as compared to patients with normal kidney function. This is unfortunate considering their increased risk of adverse outcomes and the benefit from intervention in higher CKD groups. It is therefore important to counter the notion that risk factor modification therapy should be less intensive in individuals with impaired kidney function, - rather the opposite using appropriate dose adjustments. As medication discrepancies are common in the populations with impaired function, special attention is needed to adjust risk factor modifying therapy [21].

Additionally, with decreasing kidney function, increasing attention should be put on non-traditional risk factors e.g. renal anemia, vitamin D activation, uric acid and dietary phosphate [22]. These treatments are currently usually handled by nephrologists. Although there is less solid evidence for early intervention and prevention with these risk factors, they may be future treatment targets to address also in primary care.

Our findings have several implications for primary care and the population with early, but silent increase in cardiorenal risk. As the traditional risk factors are not easily controlled, it may be of importance to start the interventions at an earlier stage. Blood pressure therapy and lipid lowering needs time to be effective and also has the potential to indirectly prevent heart failure, a risk that we find increased already in people with CKD 3. Furthermore, it is much more complicated to initiate these therapies in late-stage CKD, as there may be more side effects, hyperkalemia and less effect of lipid lowering. Fortunately, there is an overlap in risk factor therapies so that antihypertensive renin angiotensin system blocking therapies including mineralocorticoid receptor antagonists (MRAs) also prevent CKD progression and SGLT2 inhibitors have pleiotropic effects broadly reducing risk of cardiorenal events [5, 23–26]. Our data also displays a need for more broad measurement of albuminuria to more accurately determine risk in a CKD population. As seen in the large American CURE CKD, NHANES and KEEP registries [27, 28], the assessment of albuminuria is much less frequent than eGFR. However, when guideline-recommended and when in focus of primary care, as is the case regarding the care of type 2 diabetes, albuminuria sampling can reach high frequencies [29].

All citizens in Denmark have free and direct access to general practitioners, who can refer patients to biochemical testing without individual payment. In Denmark, general practitioners have a central role in the public health care system, for general health screening, chronic disease care as well as acting as gatekeepers to more specialized patient care. As our data demonstrates it would therefore be obvious to conduct future CKD screening and intervention studies in general practice. Currently, there are no recommendations for CKD screening in general practice in Denmark.

We used the KDIGO classification to diagnose CKD in our dataset. Other ways of classifying CKD in laboratory databases have been investigated in a study by Vestergaard et al. [30] where they demonstrated differences in incidence and prevalence of CKD depending on classification used. However, they found no major differences in mortality and dialysis rates.

Our study has limitations as blood pressure and smoking data were unavailable and the use of cardioprotective therapies i.e. renin-angiotensin inhibitors and statins were not included in the regression models. Very few individuals in our dataset had albuminuria measured, which is a strong risk marker, identified among others in the CKD Consortium publication [16]. The combination of eGFR and albuminuria is clearly the best, strongest and cheapest biomarkers available for risk prediction, also highly relevant for use in primary care. There is a risk of selection bias as our data is not cross sectional, and we do not have outcome information on individuals that did not have serial creatinine measurements in the dataset. We observed a low number of cases in CKD stage 5. This could be a consequence of referral of cases being referred to specialist care and thus having fewer laboratory tests performed in primary care, causing a “dilution” of the most extreme part of the population. We could however still trace clinical outcome in the national registers, minimizing the impact of migration and referral to secondary care. Completeness of outcome data, based on centralized registration of hospital admissions and death certificates is very high in Denmark, thanks to our unique personal identifier numbers. Finally, our study is observational, and as such precludes the opportunity to derive a direct cause-and-effect risk association.

In conclusion, this study in a large primary care population, demonstrates an increased risk of cardiorenal outcomes with declining kidney function, already present in individuals with CKD3. This calls for increased attention to risk factor management to prevent CKD progression and cardiovascular outcomes, just as recently proposed by the European Renal Association (www.era-online.org/en/strongkidneys/blog/do-you-know-your-abcde-profile/).

## Electronic supplementary material

Below is the link to the electronic supplementary material.

Supplementary Material 1

## Data Availability

The data that support the findings of this study are available from Statistics Denmark but restrictions apply to the availability of these data, which were used under license for the current study, and so are not publicly available. Data can only be examined in collaboration with an authorized Danish investigator. Request for access can be send to Professor Christen Lykkegaard-Andersen, christen.andersen@sund.ku.dk.

## Abbreviations

ARIC: Atherosclerosis Risk In Communities
CI: Confidence interval
CKD: Chronic kidney disease
CGPL: Copenhagen General Practitioners’ Laboratory
CVD: Cardiovascular disease
eGFR: Estimated glomerular filtration rate
ESKD: End stage kidney disease
GP: General practitioner
HbA_1c_: Haemoglobin A1c
HF: Heart failure
HR: Hazard ratio
ICD: International Classification of Disease
ISO: International Organization for Standardization
KDIGO: Kidney Diseases Improving Global Outcomes
LDL: Low density lipoprotein
MRA: Mineralocorticoid receptor antagonist
MI: Myocardial infarction
PAD: Peripheral artery disease
PIN: Personal identification number
PY: Person-years
SGLT2i: Sodium glucose cotransporter 2 inhibitor
