# Supplementary material for: Chronic kidney disease in primary care: risk of cardiovascular events, end stage kidney disease and death
Source: BMC Prim Care. 2023 Jun 21;24:128. doi: 10.1186/s12875-023-02077-7 (PMC10286349; doi:10.1186/s12875-023-02077-7)
Supplement: Supplementary file 1 — Supplementary Material 1 [file 12875_2023_2077_MOESM1_ESM.docx]

**Supplementary material**

**Supplementary Figure 1. Flowchart of the inclusion of individuals in the population.**


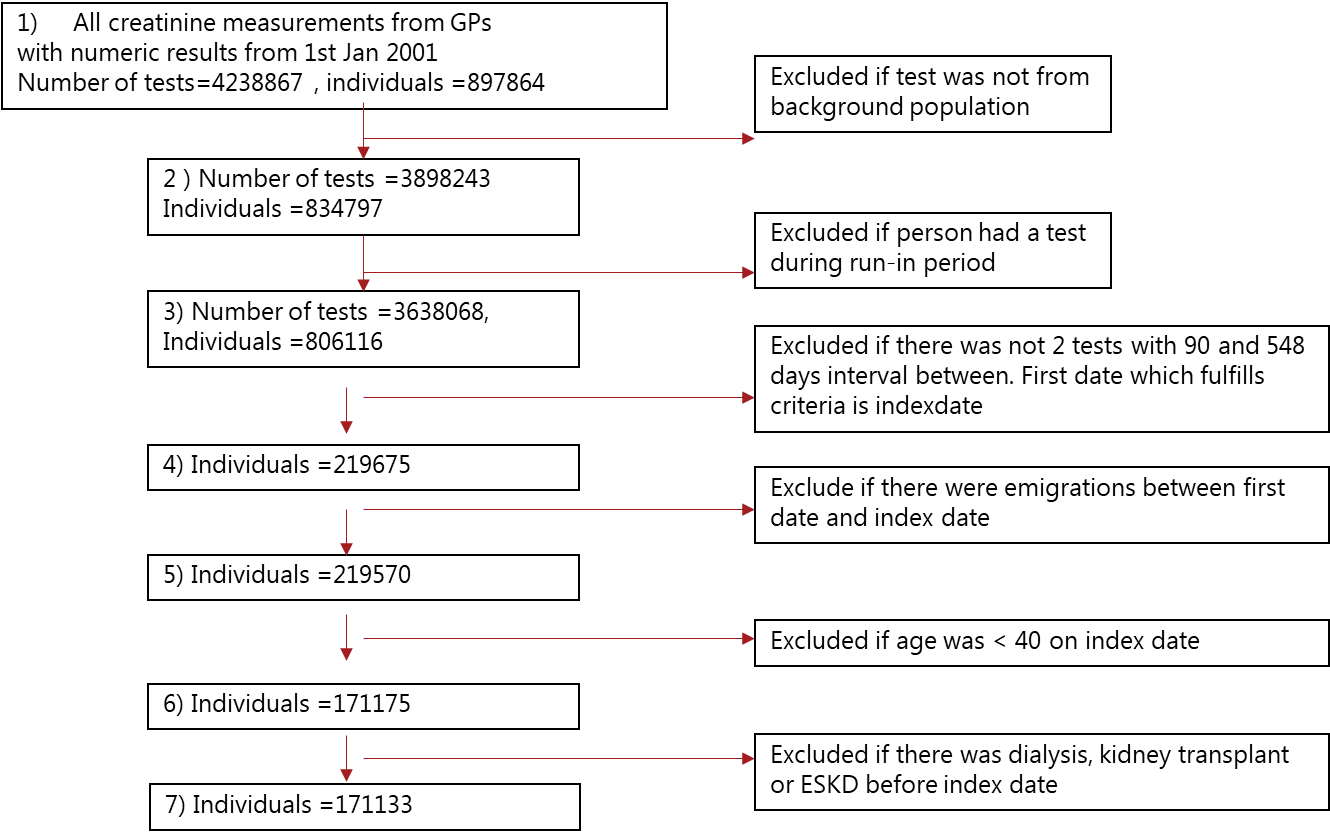


*GPs; general practitioners, ESKD; end stage kidney disease*

**Supplementary Figure 2a-g. Continuous adjusted hazard rates for all outcomes according to eGFR.**

a.


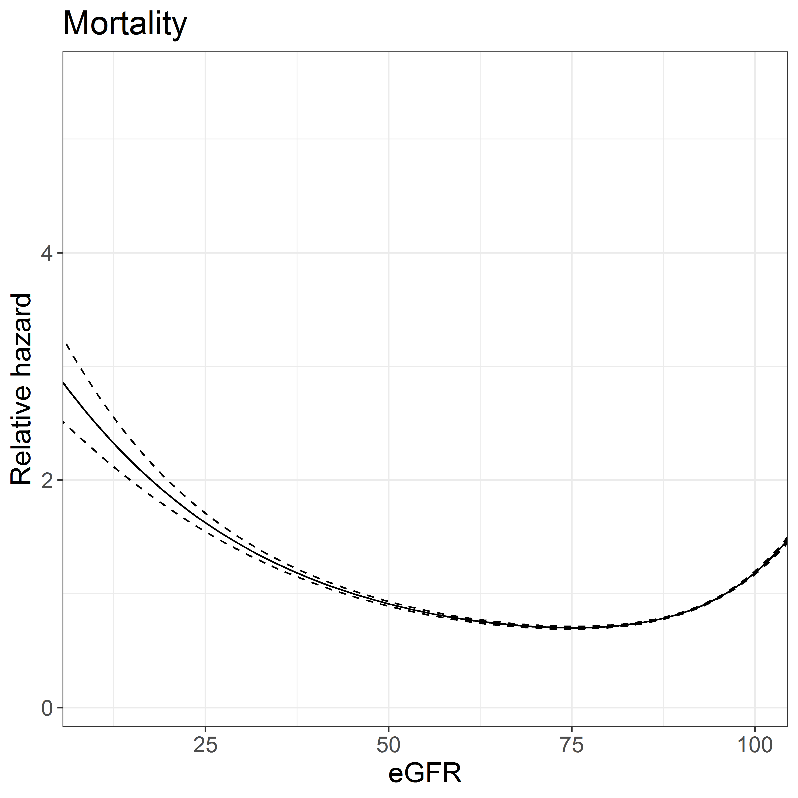


b.


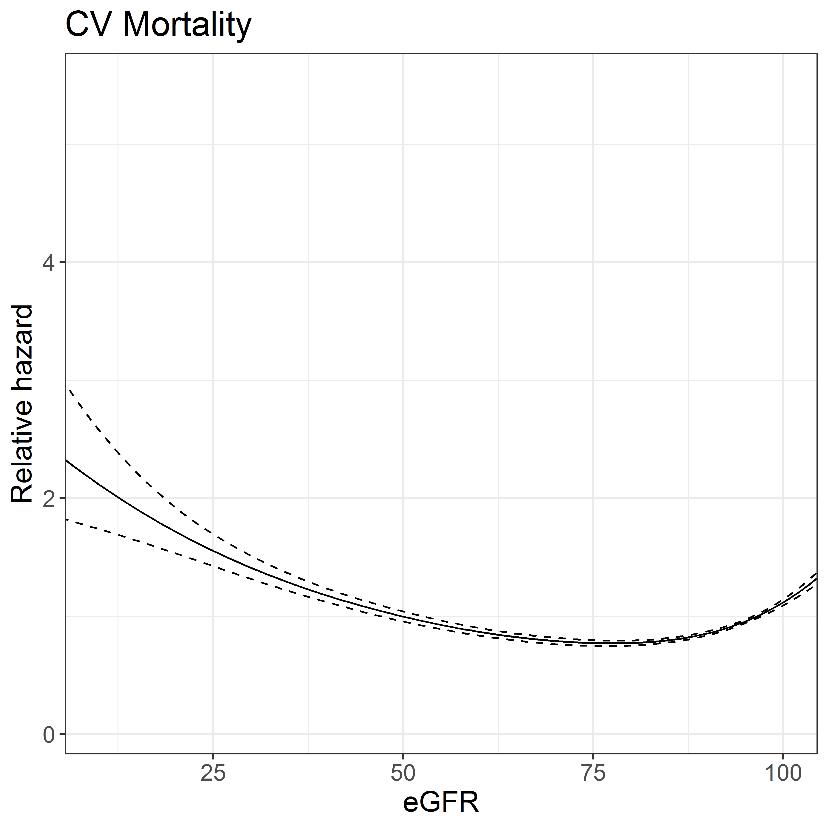


c.


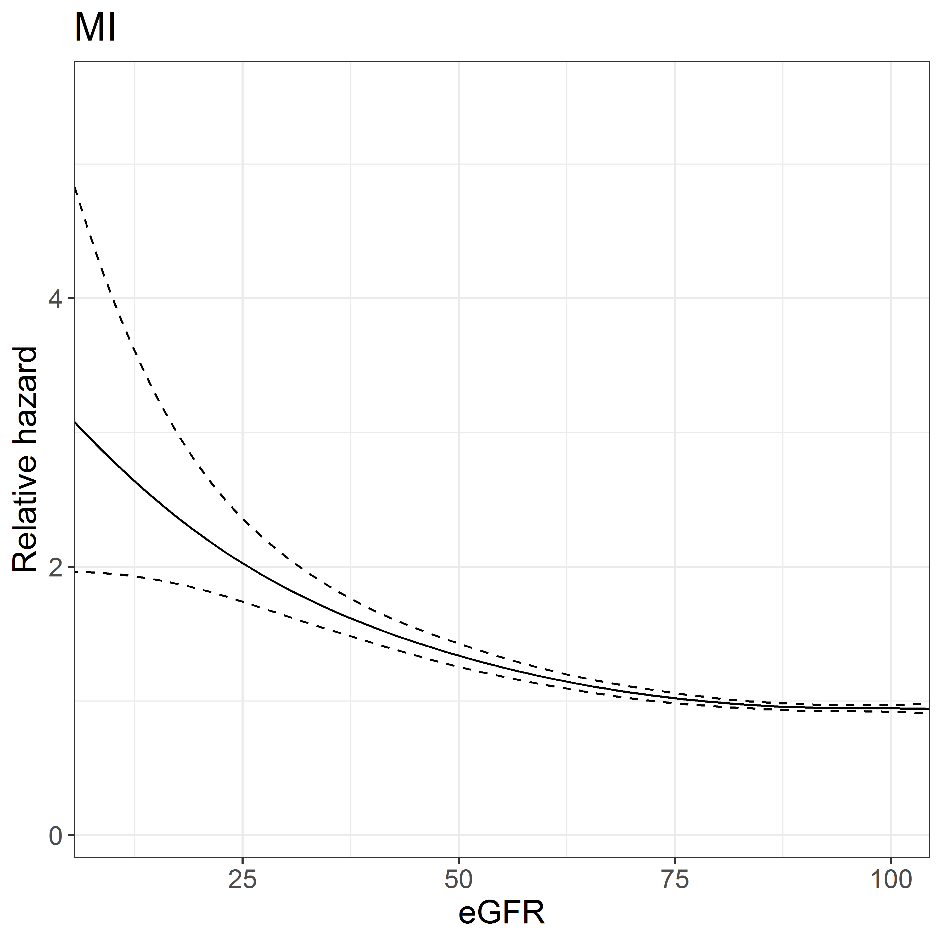


d.


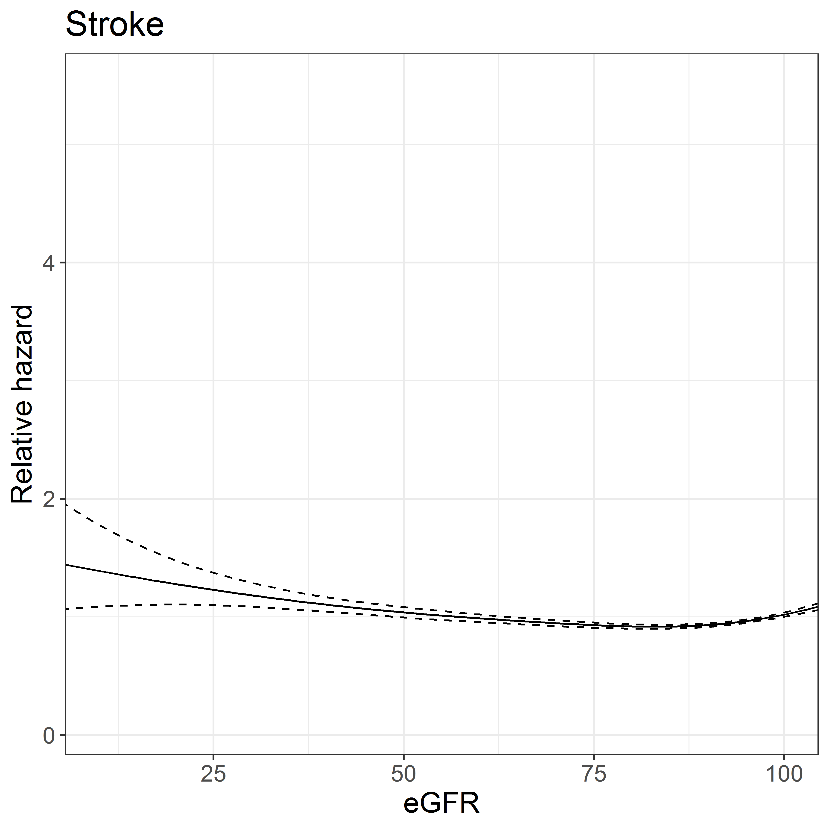


e.


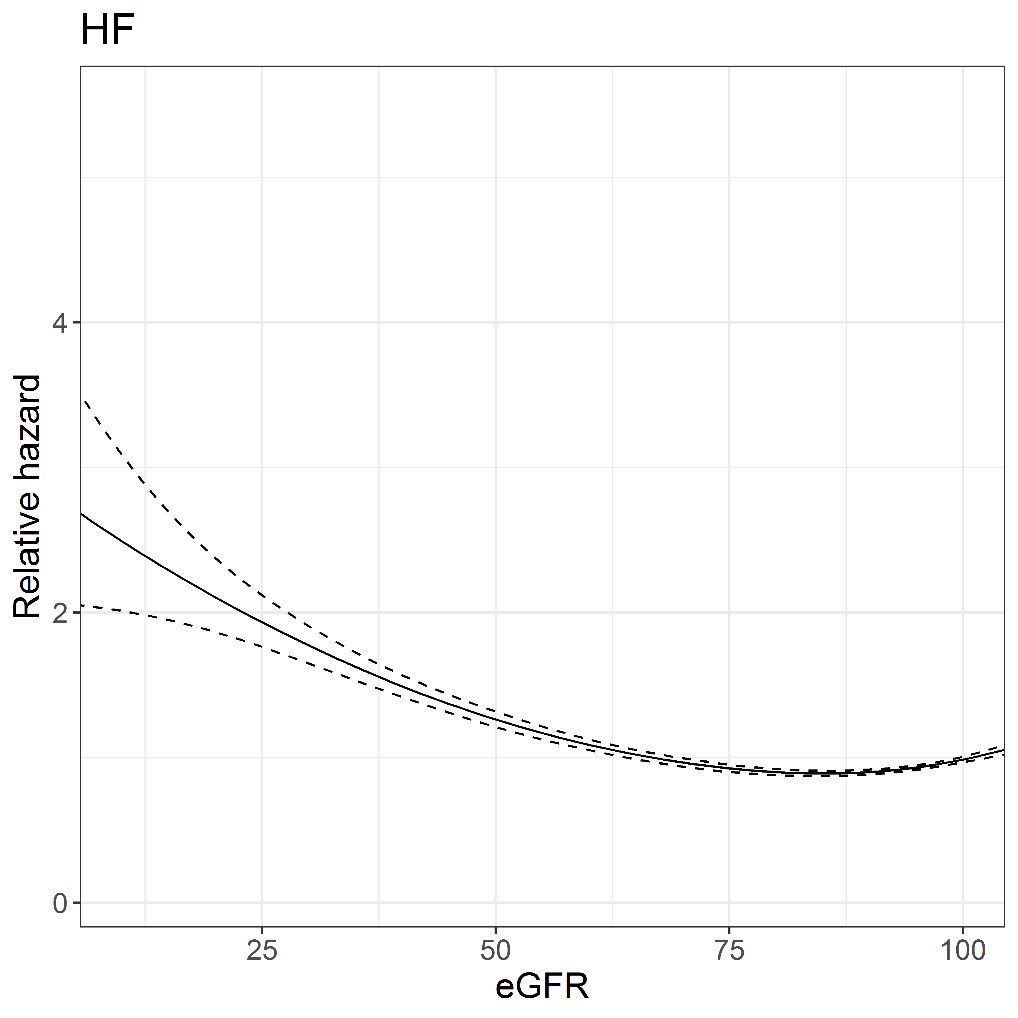


f.


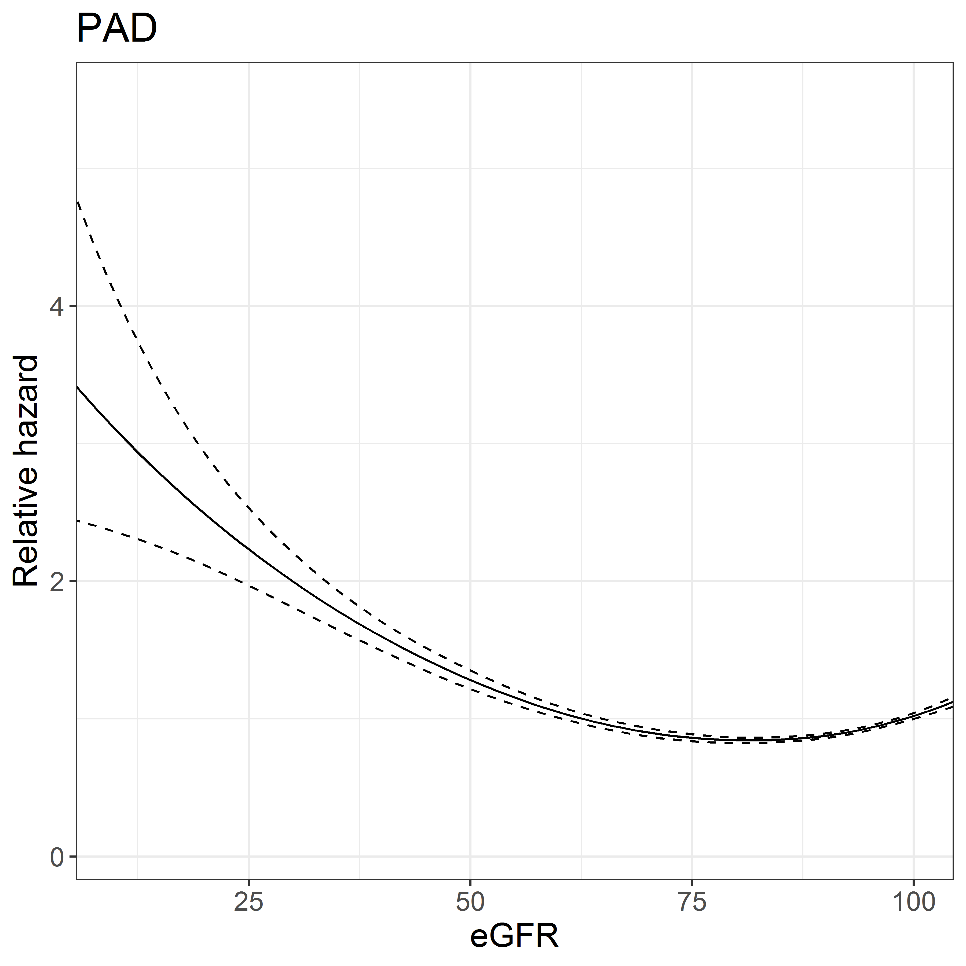


g.


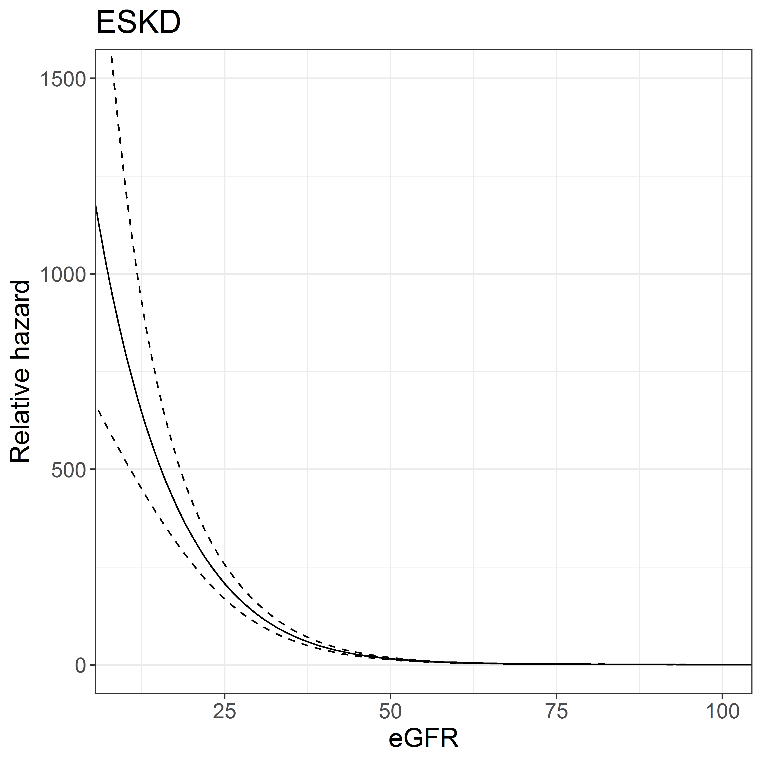


*eGFR; estimated glomerular filtration rate, CV; cardiovascular, MI; myocardial infarction, HF; heart failure, PAD; peripheral arterial disease, ESKD; end stage kidney disease.*
